# Obesity Strongly Predicts COVID-19-Related Major Clinical Adverse Events in Coptic Clergy

**DOI:** 10.3390/jcm10132752

**Published:** 2021-06-22

**Authors:** Michael Y. Henein, Ibadete Bytyçi, Rachel Nicoll, Rafik Shenouda, Sherif Ayad, Federico Vancheri, Matteo Cameli

**Affiliations:** 1 Institute of Public Health and Clinical Medicine, Umea University, 90187 Umea, Sweden; i.bytyci@hotmail.com (I.B.); rachelnicoll25@gmail.com (R.N.); rafik.shenouda@umu.se (R.S.); 2 Molecular and Clinic Research Institute, St George University, London SW17 0QT, UK; 3 Institute of Fluid Dynamics, Brunel University, London UB8 3PH, UK; 4 International Cardiac Centre, Alexandria 21526, Egypt; 5 Department of Cardiology, Faculty of Medicine, Alexandria University, Alexandria 21526, Egypt; sherifwagdyayad@yahoo.com; 6 Department of Internal Medicine, S. Elia Hospital, 93100 Caltanissetta, Italy; federico.vancheri@ki.se; 7 Department of Medical Biotecnologies, Division of Cardiology, University of Siena, 53100 Siena, Italy; matteo.cameli@yahoo.com

**Keywords:** COVID-19, Coptic clergy, prevalence, major adverse events, obesity

## Abstract

Background and Aims: The Coptic clergy, due to their specific work involving interaction with many people, could be subjected to increased risk of infection from COVID-19. The aim of this study, a sub-study of the COVID-19-CVD international study of the impact of the pandemic on the cardiovascular system, was to assess the prevalence of COVID-19 among Coptic priests and to identify predictors of clinical adverse events. Methods: Participants were geographically divided into three groups: Group-I: Europe and USA, Group II: Northern Egypt, and Group III: Southern Egypt. Participants’ demographic indices, cardiovascular risk factors, possible source of infection, number of liturgies, infection management, and major adverse events (MAEs), comprising death, or mechanical ventilation, were assessed. Results: Out of the 1570 clergy serving in 25 dioceses, 255 (16.2%) were infected. Their mean age was 49.5 ± 12 years and mean weekly number of liturgies was 3.44 ± 1.0. The overall prevalence rate was 16.2% and did not differ between Egypt as a whole and overseas (*p* = 0.23). Disease prevalence was higher in Northern Egypt clergy compared with Europe and USA combined (18.4% vs. 12.1%, *p* = 0.03) and tended to be higher than in Southern Egypt (18.4% vs. 13.6%, *p* = 0.09). Ten priests (3.92%) died of COVID-19-related complications, and 26 (10.2) suffered a MAE. The clergy from Southern Egypt were more obese, but the remaining risk factors were less prevalent compared with those in Europe and USA (*p* = 0.01). In multivariate analysis, obesity (OR = 4.180; 2.479 to 12.15; *p* = 0.01), age (OR = 1.055; 0.024 to 1.141; *p* = 0.02), and systemic hypertension (OR = 1.931; 1.169 to 2.004; *p* = 0.007) predicted MAEs. Obesity was the most powerful independent predictor of MAE in Southern Egypt and systemic hypertension in Northern Egypt (*p* < 0.05 for both). Conclusion: Obesity is very prevalent among Coptic clergy and seems to be the most powerful independent predictor of major COVID-19-related adverse events. Coptic clergy should be encouraged to follow the WHO recommendations for cardiovascular disease and COVID-19 prevention.

## 1. Introduction

COVID-19 is an aggressive pandemic that has claimed the life of millions worldwide [1]. Virus mutations, a known phenomenon, have now been detected in Europe, Africa, and America, carrying with it doubt about the protective effect of the recently developed vaccines [2,3]. According to the World Health Organization (WHO) recommendations, optimum personal hygiene and physical distancing are the two most important means of preventing virus transmission; hence, the internationally implemented strict lockdowns represent a life-saving strategy despite their drastic impact on the world’s economy [4,5].

Coptic priesthood is considered a life vocation with no retirement and with a heavy burden of service involving liturgies, church meetings, home visits, etc. [6]. Furthermore, the nature of the liturgical services requires close contact in the form of touching hands, kissing crosses and icons, and sharing sacramental vessels, a practice that subjects the Coptic clergy to potential infection risk [7,8].

The aim of this study was to assess the prevalence of Coptic clergy who caught COVID-19, irrespective of the clinical management regimen required, home treatment, or hospital admission, to identify the potential risk factors that contributed to disease spread, and to propose practical means for optimum disease prevention.

## 2. Methods

This is a sub-study within the COVID-19-CVD international study, which investigates the impact of the pandemic on the cardiovascular system, and which has been approved by the Swedish Ethics Board (Dnr 2020-02217 Stockholm avdelning 2 medicin) and the International Cardiac Centre-ICC Ethics Board (ICC, 3/2021, Egypt). M.Y.H. (The Principal Investigator-PI) designed the study protocol, which was endorsed by the Head of the Coptic Church in Egypt. The anonymized data were sent to I.B., a Ph.D. candidate, for statistical analysis.

**Figure 1:**
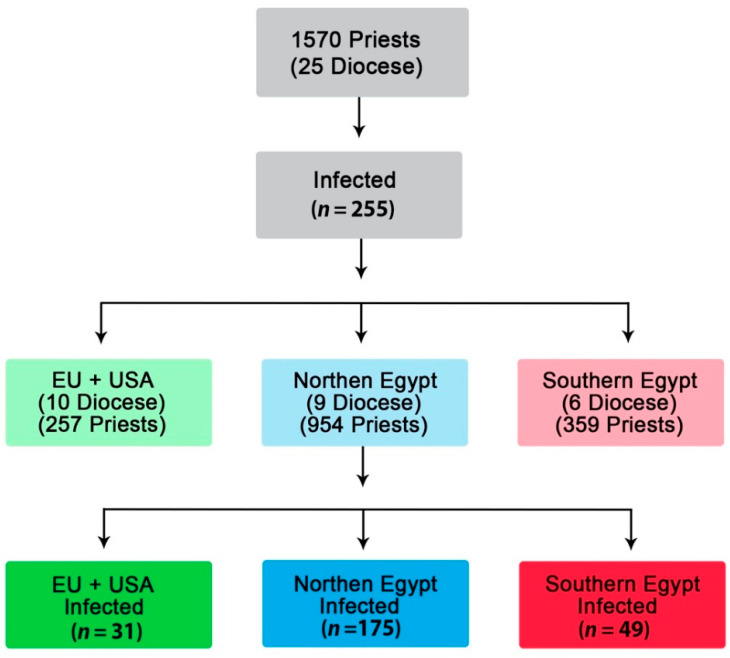
Flow chart of participants.

Since weather conditions and individual habits differ between various regions of Egypt, with some humid (North) and others hot and dry (South), the potential impact of geographic distribution on the prevalence of infection in Egypt and abroad (Europe and the USA) was also assessed (Figure 1). Dioceses in Egypt were divided into two main regions, North, including Alexandria, Delta, and Cairo; and South, including all cities geographically south of Cairo. Data collected from European countries and the USA were presented and analyzed, having been combined together in one group since they mostly followed similar disease prevention and treatment methods, including social distancing. We did not have data on uninfected and asymptomatic but spreadable clergy to compare with infected clergy. The study duration was from March till December 2020.

### 2.1. Clinical Events

Clinical events (CE) were retrospectively collected while compiling data from dioceses. Information on participants’ clinical outcomes were obtained from the medical records, clinical visits, personal communication with general physicians, and telephone interviews with patients and relatives, in a strict confidential way. The primary study end-point was major adverse events (MAEs), defined as the combination of death related to COVID-19 and mechanical ventilation. The secondary clinical outcomes were death, mechanical ventilation, or re-infection.

### 2.2. Cardiovascular Risk Factors Assessment

Participants’ conventional cardiovascular risk factors were assessed as follows: overweight was determined as body mass index (BMI) of 25–29.9 kg/m^2^, and obesity was taken as BMI ≥ 30 kg/m^2^. Systemic hypertension (AH) was diagnosed when systolic blood pressure (SBP) was ≥130 mmHg and/or diastolic blood pressure (DBP) was ≥80 mmHg or when antihypertensive therapy was prescribed. Diabetes mellitus (DM) was considered based on pre-recruitment diagnosis leading to participants who commenced conventional oral hypoglycemics and/or insulin therapy. Hypercholesterolemia was determined from medical records or if he had been prescribed statins. Evidence for coronary artery disease was also gathered from medical records, based on prior investigations and management.

### 2.3. Statistical Analysis

Data are summarized using frequencies (percentages) for categorical variables and mean ± standard deviation (SD) for continuous variables or median interquartile (IRQ) ranges. Continuous data were compared with two-tailed Student *t* test and discrete data with chi-square test. Analysis of variance and Bonferroni statistical tests were used to compare quantitative variables between more than two groups. The degree of association between clinical variables and disease prevalence was determined using the Pearson’s correlation coefficient in the case of continuous variables; chi-square test (categorical and categorical variables) and point biserial correlation were used in the case of categorical and continuous variables. Predictors of clinical complications (mechanical ventilation) and death were identified with univariate analysis. Independent predictors were identified using multivariate logistic regression analysis using the stepwise method. A significant difference was defined as *p* value < 0.05 (2-tailed). Statistical analysis was performed with SPSS Software Package version 26.0 (IBM Corp., Armonk, NY, USA).

## 3. Results

### 3.1. Demographic Indices of the Participating Clergy

Twenty-five dioceses provided data on their affected clergy: 15 from Egypt and 10 from overseas (Europe and USA). Out of the 1570 clergy with available data, 255 (16.2%) were infected with the following likely source of infection: church (30.1%), home (12.8%), and personal contact (16.3%), while the remaining (40.1%) were unknown. The mean age of infected clergy was 49.5 ± 12 years, and the mean weekly number of served liturgies was 3.44 ± 1.0. Two hundred and ten (82.7%) of the infected clergy were treated at home, and the remaining forty-four (17.3%) required hospital admission and management; sixteen (6.72%) of them needed mechanical ventilation. Ten clergy (3.92%) died of COVID-19-related complications, and twenty-six (10.2%) of the infected clergy suffered major adverse events (Table 1).

### 3.2. Impact of Cardiovascular Risk Factors on Disease Prevalence

**Figure 2:**
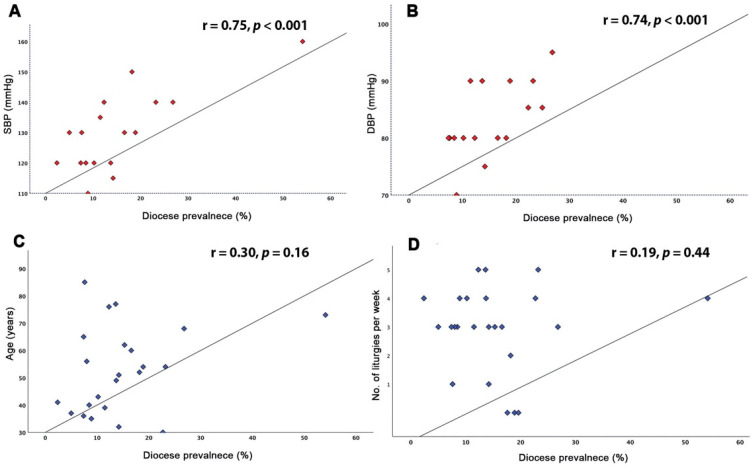
Relationship between risk factors and prevalence of COVID-19 among Clergy. (**A**) Disease prevalence with SBP; (**B**) disease prevalence with DBP; (**C**) disease prevalence with age; (**D**) disease prevalence with number of liturgies per week. SBP: systolic blood pressure; DBP: diastolic blood pressure.

**Figure 3:**
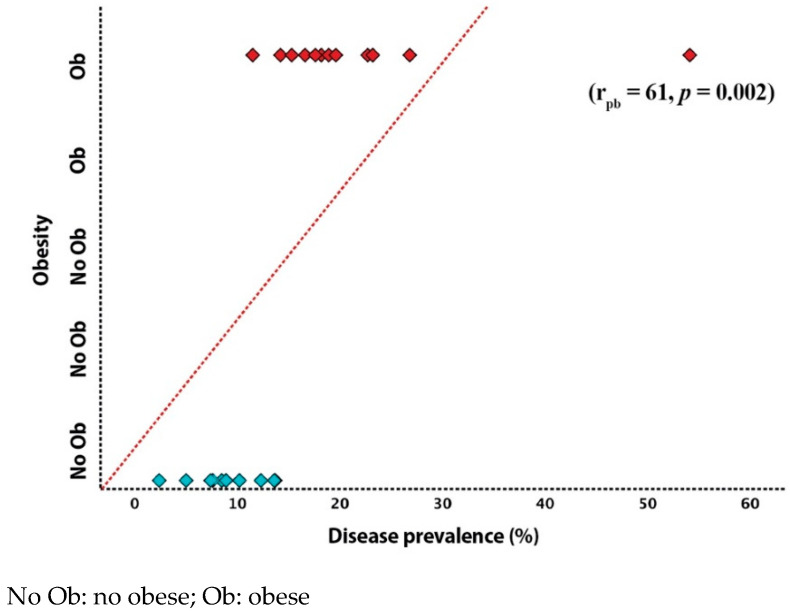
Relationship between obesity and prevalence of COVID-19 among Clergy.

One hundred and one (39.8%) clergy were overweight, and one hundred and thirty-three (52.2%) were obese. Seventy-one (27.9%) clergy were on anti-hypertensive medications, sixty (23.5%) were treated for diabetes, sixty-seven (26.5%) were treated for dyslipidemia, and twenty-four (9.6%) had a history of coronary artery disease (Table 1). Hypertension measurements, taken at the time of data collection, correlated strongly with the prevalence of COVID-19, SBP (r = 0.78, *p* < 0.001), and DBP (r = 0.74, *p* < 0.01, Figure 2). Similarly, obesity strongly correlated with disease prevalence (r_pb_ = 0.61, *p* = 0.002, Figure 3). No relationship was found between age, BMI, diabetes, or number of weekly served liturgies and the prevalence of COVID-19 (*p* > 0.05 for all, Table 2). We also tested the possible impact of dioceses with higher disease prevalence on the correlation analysis. In influence analysis, the relationship between diocese prevalence and SBP, DBP, age, and number of liturgies per week showed almost similar correlation, with only small reduction of magnitude with SBP and DBP (Supplementary Materials Figure S1).

### 3.3. Geographical Impact on Disease Prevalence

**Figure 4:**
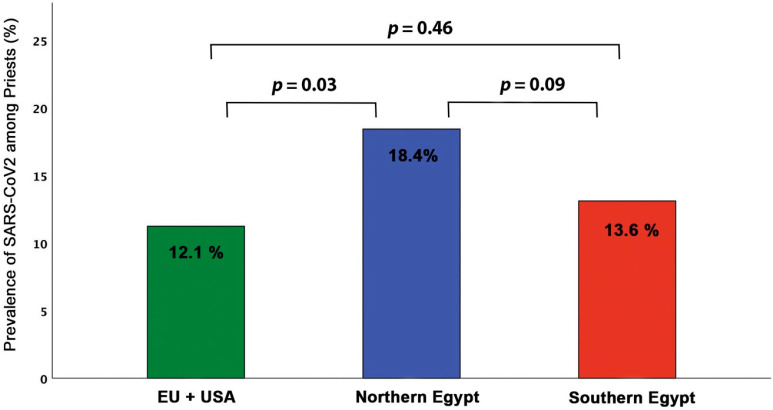
Prevalence of COVID-19 among Clergy in different regions.

The overall prevalence of COVID-19 infection among clergy did not differ between Egypt, as a whole and abroad (*p* = 0.23). The Northern Egypt clergy had significantly higher disease prevalence compared with the regions of Europe and USA combined (18.4% vs. 12.1%, *p* = 0.03) and tended to be higher compared with Southern Egypt (18.4% vs. 13.6%, *p* = 0.09). There was no difference in disease prevalence between Southern Egypt and Europe and USA combined (*p* = 0.46, Figure 4).

We also tested the impact of any dioceses with higher disease prevalence on the overall prevalence of the region. Five of the twenty-five dioceses had high prevalence (above 18%, Supplementary Materials Figure S2): two from Northern Egypt, two from Southern Egypt, and one from the Europe and USA combined group, compared with the remaining dioceses. Obesity tended to be more prevalent (*p* = 0.052) in those five dioceses combined, but the rest of the CV risk factors and demographic indices were not significantly different compared with the rest of the dioceses (*p* > 0.05 for all). Testing the influence analysis, the overall mean between regions proved insignificant (10.8%, 12.9%, 9.2%, respectively, *p* > 0.05 for all), suggesting no significant individual diocesan influence.

In subgroup analysis based on the region where clergy lived and served, no difference was found by subject age, BMI, systolic or diastolic blood pressure, and number of weekly served liturgies (*p* > 0.05 for all) between regions. The clergy from Southern Egypt were more obese compared with those in Europe and USA (*p* = 0.01), but other risk factors were less prevalent compared with Northern Egypt and compared with the combination of Europe and the USA: hypertension (24.3%, 38%, and 36.4%; *p* < 0.05, respectively) and coronary heart disease (2.8%, 11.3%, 9.5%; *p* < 0.05, respectively). The prevalence of diabetes and dyslipidemia was not different between groups according to location (Table 3).

### 3.4. Predictors of Clinical Events

The overall mortality rate in the study participants was 4.42%, with no difference between Northern and Southern Egypt (4.1% vs. 6.4%, *p* > 0.05) and with 0% mortality in Europe and USA. Likewise, the rate of MAE was 2 times higher in the two Egyptian regions compared with Europe and the USA (12.6% vs. 6.4%, *p* < 0.001). MAEs were significantly higher in Southern Egypt clergy compared with the Northern Egypt clergy (14.9% vs. 10.2%, *p* < 0.05 Table 3).

In univariate analysis, age (*p* = 0.003), CHD (*p* = 0.002), hospital treatment (*p* = 0.001), and mean number of days of home treatment (*p* = 0.001) predicted mortality. In a multivariate analysis model, only hospital treatment (OR 3.116; 2.586 to 4.796; *p* = 0.007) predicted COVID-19 disease-related mortality (Table 4). Age (*p* = 0.02), obesity (*p* = 0.02), CHD (*p* = 0.03), mean days of home treatments (*p* = 0.04), and arterial hypertension (*p* = 0.01) were all predictors of combined MAEs (death and mechanical ventilation), but in multivariate analysis, the independent predictors of MAE were: obesity (OR = 4.180; 2.479 to 12.15; *p* = 0.01), age (OR = 1.055; 0.024 to 1.141; *p* = 0.01), and arterial hypertension (OR = 1.931; 1.169 to 2.004; *p* = 0.007). Testing the impact of geographical distribution on the predictors of COVID-19-related MAE showed that obesity was the most powerful independent predictor in Southern Egypt, and systemic hypertension was the most powerful independent predictor in Northern Egypt. Because of the limited data available, we could not test the MAE in regions of Europe and USA (Table 5). Collinearity between these measurements was not met based on VIF < 10 for all predictors.

### 3.5. Comparison with Data from other Communities

Churches around the world had serious loss of life as a result of the pandemic [9], but available official data are very limited. Our results show that the overall prevalence of COVID-19 among Coptic clergy was 14.2% (18.4%, 13.4%, 12.1% for Northern Egypt, Southern Egypt, and combined Europe and USA, respectively). This prevalence is significantly higher than that reported in the UK (7%, *p* < 0.001). Compared with other communities with somewhat shared traditions, Coptic Egypt demonstrated less disease prevalence in the Coptic clergy compared with ultra-Orthodox Jews (constituting 10–12% of inhabitants of Israel) in whom the disease prevalence proved to be 40% [10]. Even in the UK, the disease prevalence rate among ultra-Orthodox Jews in London was reported to be significantly high, approaching 64%, compared with the overall country prevalence of 7% (*p* < 0.001) [11]. Similar comparisons apply to the overall COVID-19-related mortality rate of Italian Catholic clergy, which proved to be significantly lower than in our Coptic clergy [12] in Northern and Southern Egypt (0.66% vs. 4.72% vs. 8.1%, respectively, *p* < 0.05).

## 4. Discussion

**Findings:** The findings of this paper can be summarized as showing significantly higher prevalence of COVID-19 infection and mortality rate among Coptic clergy compared with other clergy from overseas and compared with other communities, irrespective of their religion and practices. Furthermore, the two most important risk factors that predicted major clinical adverse events were overweight/obesity and hypertension, although these risk factors were less prevalent in clergy serving in Europe and USA, compared with those serving in Egypt.

**Data interpretation:** Our three important findings are not independent of each other. Obesity is a recognized risk factor for COVID-19 infection and is associated with poor survival, particularly among patients treated in intensive care units and those requiring mechanical ventilation [13,14]. Obesity is known to be associated with a compromised immune system [15], in addition to the significant buildup of internal body fat, which has its mechanical effect in compromising normal physiological respiration including diaphragmatic and lung function [16,17]. Superspreading events (where, typically, 20% of those infected account for 80% of virus transmission) are triggered by increased exhalation of droplets deriving from airway mucosal surfaces during respiration due to degraded airway lining mucous barrier function. A recent study showed that aerosol exhalation increased with severity of COVID-19 infection and higher elevated BMI-years (BMI multiplied by age), with 18% of subjects, who were both older and with higher BMI, accounting for 80% of the total exhaled bioaerosols [18]. These consequences of overweight and obesity explain the high rate of infection and mortality in the studied Coptic clergy. The second most important predictor of infection was hypertension; again, hypertension has long been known for its effect on cardiac structure and function, as well as kidney function, with resulting complications and compromised body immunity [19,20]. On the other hand, diabetes patients, particularly type 1 patients, are prone to worse outcomes when infected with SARS-CoV2, similar to other respiratory viruses, hence indicating an urgent need to mitigate severe acute respiratory syndrome CoV2 infection risk in this group of patients [21,22,23]. Because of the low prevalence of diabetes in our study population, particularly type 1 diabetes (2.65%), diabetes was not a key risk factor in predicting major adverse events.

Our results show that COVID-19 disease prevalence and related mortality among Coptic clergy are significantly high compared with clergy from other denominations and also compared with other communities, irrespective of similar cultural habits and geographical location. This should be taken seriously on the basis of the other contributing factors. The Coptic clergy lifestyle is significantly different from that of other clergy and ordinary individuals. Their work is commonly in crowded places, churches, and meeting rooms, and they make many home visits. It also involves frequent touching of sacramental vessels, cloths, crosses, icons, etc. According to the WHO recommendations for the pandemic prevention strategy, such an extent of material/object touching is against all health and sanitary advice and could be explained as a potential cause of infection [3,24]. Furthermore, Coptic clergy have the tradition of growing long beards, which can conceivably be seen as a source of continuous infection, being in close proximity to the priest’s breath and salivary droplets while talking or giving a sermon. Furthermore, clergymen frequently tough their beards before greeting others. Adding to this, while the WHO recommends frequent thorough hand washing, this cannot be optimally adhered to because of the long hours clergy spend away from home in service to the community. Finally, the social habits and practices are likely to have played a major role, but in the absence of robust evidence for that we felt reluctant to overemphasize it. The latter point is supported by the lower rate of infection and mortality, as well as the lower prevalence of COVID-19 complications in Coptic clergy serving in Europe and USA, where they abide by local rules, as compared to those serving in Egypt.

The findings of the geographical distribution analysis show significantly higher disease prevalence in Northern Egypt compared with Southern Egypt, with obesity as the only independent predictor of MAE in the south and hypertension as the only independent predictor in the north [25,26]. This highlights the importance of local regional community habits and means of undertaking services. Climate may also have a bearing, with the lower prevalence being found in Southern Egypt, where it is hotter, reflecting the findings of other studies. It is well known that high heat reduces the spread of COVID-19 and that the reduction is assisted by high humidity, as reflected in a recent systematic review [27]. High humidity is a factor in determining COVID-19 infectivity, as dry air is known to enable viral transmission and is associated with impaired mucociliary clearance, innate antiviral defense, and tissue repair function. In this instance, the higher heat of Southern Egypt seems to be a greater factor than the higher humidity of the north [28]. In addition, obesity was less prevalent in Coptic clergy serving in Europe and USA, where there was zero COVID-19-related mortality, suggesting better awareness of disease prevention and healthier lifestyle. Finally, the five dioceses identified with very high (18%) prevalence of infection were evenly distributed in the three regions, which strengthens the relevance of lifestyle impact and the customary habits of Coptic clergy having an additional risk for COVID-19 infection and related mortality.

**Clinical implications:** Obesity is the major risk factor for COVID-19-related major clinical adverse events, including mortality. Significant changes of lifestyle and dietary habits need to be adopted by Coptic clergy in order to maintain general body health and protect them from other related conditions, e.g., hypertension and diabetes. The strong predictive value of obesity for major clinical adverse events highlights its importance, irrespective of the presence of diabetes, the more commonly investigated disease, suggesting the urgent need for their vaccination to guarantee better prevention.

**Limitations:** This was a retrospective study, so the amount of data available for analysis was limited. We did not have data on uninfected and asymptomatic but spreadable clergy, which would have provided a more accurate and meaningful comparison. Detailed information on clinical care, blood analyses, and accurate assessment of the means of infection were based on the data provided by the clergy themselves rather than using a centralized approach. The small sample size did not allow us to test the impact of geographical distribution among Coptic clergy in Europe and USA. Not all dioceses complied with the request to provide data, so we relied on the available information from 25 dioceses only to run the statistical analyses and document the results for the benefit of Coptic and other clergy. PCR testing was not available in all clergy, but diagnosis and treatment was designed based on clinical findings and antibody detection.

**Conclusions:** Obesity is very prevalent among Coptic clergy and seems to be the most powerful independent predictor of major COVID-19-related adverse events. Furthermore, because of the nature of their lifestyle, Coptic clergy represent a high-risk group for COVID-19, highlighting the need for stringent management of cardiovascular risk factors according to the well-established WHO recommendations.

## Figures and Tables

**Table 1:** Demographic, clinical, and outcome data of Coptic clergy.

Variable	Priests (*n* = 255)
**Demographic and clinical data**	
Age (years)	49.5 ± 12
BMI (m/kg^2^)	32 ± 6.2
SBP (mmHg)	127 ± 13
DBP (mmHg)	83 ± 9.5
Underweight (*n*, %)	0 (0)
Normal weight (*n*, %)	21 (8.4)
Overweight (*n*, %)	101 (39.8)
Obese (*n*, %)	133 (52.2)
AH (*n*, %)	71 (27.9)
DM (*n*, %)	60 (23.5)
DM type 1 (*n*, %)	6 (2.65%)
Dyslipidemia	67 (26.5)
CHD (*n*, %)	24 (9.6)
Family history for CHD (*n*, %)	22 (8.9)
Family history for stroke (*n*, %)	14 (5.8)
Liturgies per week	3.44 ± 1.0
Source of infection	
Home (*n*, %)	32 (12.8)
Church (*n*, %)	76 (30.1)
Personal (*n*, %)	41 (16.3)
Unknown (*n*, %)	102 (40.1)
**Outcome data**	
Home treatment (*n*, %)	210 (82.7)
Hospital treatment (*n*, %)	44 (17.3)
Intensive care (*n*, %)	21 (8.4)
Home treatment (days)	17.9 ± 10.3
Hospital treatment (days)	10.2 ± 9.4
Intensive care (days)	7.4 ± 3.4
Mechanical ventilator (*n*, %)	16 (6.72)
Death (*n*, %)	10 (3.92)
MAE (*n*, %)	26 (10.2)

AH: arterial hypertension; BMI: body mass index; CHD; coronary heart disease; DM: diabetes mellitus; SBP: systolic blood pressure; DBP: diastolic blood pressure.

**Table 2:** Relationship between prevalence with demographic and clinical variables.

Variable	r	*p* Value
Age	0.30	0.16
BMI	−0.12	0.57
CHD	−0.38	0.09
DM	0.10	0.68
SBP	0.75	<0.001
DBP	0.74	<0.001
Obesity	0.61	0.002
Liturgies per week	0.19	0.44

BMI: body mass index; CHD: coronary heart disease; DM: diabetes mellitus; SBP: systolic blood pressure; DBP: diastolic blood pressure.

**Table 3:** Demographic, clinical and outcome data of priests in different regions.

Variable	EU+ USA (*n* = 31)	Northern Egypt (*n* = 175)	Southern Egypt (*n* = 49)	*p*-Value
**Demographic and clinical data**				
Age (years)	52.7 ± 11	49.6 ± 12	47.4± 11	NS
BMI (m/kg^2^)	31 ± 9.1	32 ± 5.7	33 ± 5.3	NS
SBP (mmHg)	126 ± 10	127 ± 14	125 ± 11	NS
DBP (mmHg)	82 ± 6.1	83 ± 9.6	84 ± 11	NS
Underweight (*n*, %)	0 (0)	0 (0)	0 (0)	NS
Normal weight (*n*, %)	5 (15.4)	10 (6.10) ^**a**^	5 (11.5)	0.03
Overweight (*n*, %)	16 (51.6)	72 (41.2) ^**a**^	12 (25.5) ^**b,c**^	0.04
Obese (*n*, %)	10 (30.7)	92 (52.6) ^**a**^	29 (59.5) ^**b**^	0.02
AH (*n*, %)	11 (36.4)	66 (38)	12 (24.3) ^**b,c**^	0.001
DM (*n*, %)	9 (28.6)	46 (26.6)	14 (29.7)	NS
Dyslipidemia	9 (31.8)	60 (34.6) ^**a**^	10 (22.2) ^**c**^	0.03
CHD (*n*, %)	3 (9.5)	20 (11.36)	1 (2.85) ^**b,c**^	0.004
Family history of CHD (*n*, %)	7 (23.8)	19 (10.8)	0 (0) ^**b,c**^	0.01
Family history of stroke (*n*, %)	3 (10.7)	10 (5.92)	9 (4.4) ^**b**^	0.04
Liturgies per week (*n*, %)	3.6 ± 1.2	3.4 ± 1.0 ^**a**^	3.5 ± 0.8	NS
Source of infection				
Home (*n*, %)	3 (9.7)	20 (11.7)	13 (28.2) ^**b,c**^	0.02
Church (*n*, %)	11 (35.5)	54 (30.9)	15 (32.6)	NS
Personal (*n*, %)	5 (16.1)	32 (18.4)	7 (15.2)	NS
Unknown (*n*, %)	12 (40.1)	68 (38.9)	11 (23.9) ^**b,c**^	0.001
**Outcome data**				
Home treatment (*n*, %)	27 (88)	129 (74.2)	46 (97.7) ^**c**^	0.04
Hospital treatment (*n*, %)	10 (31.8)	26 (15.1) ^**a**^	8 (18.4)	0.03
Intensive care (*n*, %)	3 (8.7)	19 (10.8)	5 (10.2)	NS
Home treatment (days)	17.2 ± 11	18.1 ± 11	17.7 ± 6.9	NS
Hospital treatment (days)	12.5 ± 12	9.7 ± 6.4 ^**a**^	8.6 ± 4.5	0.001
Intensive care (days)	4.2 ± 3.9	6.9 ± 3.2	13 ± 8.4 ^**a**^	0.01
Mechanical ventilator (*n*, %)	2 (4.8)	10 (5.71)	4 (8.2)	NS
Death (*n*, %)	0 (0)	7 (4.1) ^**a**^	3 (6.4) ^**b**^	<0.001
MAE (*n*, %)	2 (6.4)	17 (10.2) ^**a**^	7 (14.9) ^**b,c**^	<0.001

^a^ *p* < 0.05; Gr. I vs. II, ^b^ *p* < 0.05; Gr. I vs. III, ^c^ *p* < 0.05; Gr. II vs. III. AH: arterial hypertension; BMI: body mass index; CHD: coronary heart disease; DM: diabetes mellitus; SBP: systolic blood pressure; DBP: diastolic blood pressure; NS: non significant.

**Table 4:** Predictors of death among infected clergy.

Variable	Univariate Predictors OR (95% CI)	*p*-Value	Multivariate Predictors OR (95% CI)	*p*-Value
Age	1.085 (1.029 to 1.153)	0.003	1.001 (0.918 to 1.092)	0.98
BMI	1.022 (0.925 to 1.130)	0.66		
Diabetes	0.745 (0.045 to 3.706)	0.71		
Obesity	5.461 (1.015 to 16.94)	0.06		
AH	0.511 (0.103 to 2.530)	0.41		
Dyslipidemia	1.070 (0.257 to 4.014)	0.81		
CHD	1.429 (1.271 to 3.144)	0.002	3.007 (0.282 to 6.059)	0.36
No. of liturgies	0.800 (0.415 to 1.541)	0.51		
Home treatment	1.910 (0.232 to 15.74)	0.54		
Hospital treatment	4.615 (3.836 to 5.958)	0.001	3.116 (2.586 to 4.796)	0.007
Mean home days	0.784 (0.671 to 0.915)	0.002	0.922 (0.803 to 1.059)	0.25
Mean hospital days	1. 010 (0.928 to 1.100)	0.21		

AH: arterial hypertension; BMI: body mass index; CHD: coronary heart disease; DM: diabetes mellitus; SBP: systolic blood pressure; DBP: diastolic blood pressure.

**Table 5:** Predictors of MAE among infected clergy.

Variable	Univariate Predictors OR (95% CI)	*p*-Value	Multivariate Predictors OR (95% CI)	*p*-Value
Age	1.031 (0.991 to 1.008)	0.04	1.055 (0.024 to 1.141)	0.01
AH	1.938 (1.172 to 2.001)	0.01	1.931 (1.169 to 2.004)	0.007
Diabetes	0.702 (0.222 to 2.170)	0.52		
BMI	1.011 (0.901 to 1.209)	0.26		
Obesity	3.366 (1.055 to 9.785)	0.02	4.180 (2.479 to 12.15)	0.01
Dyslipidemia	0.710 (0.312 to 2.231)	0.55		
CHD	4.122 (1.202 to 15.01)	0.02	3.625 (0.802 to 17.89)	0.09
No. of liturgies	0.608 (0.451 to 1.342)	0.31		
Home days	0.997 (0.806 to 1.011)	0.04	1.480 (0.209 to 7.032)	0.62
Hospital days	0.990 (0.801 to 1.068)	0.33		
**Northern Egypt**				
Age	1.081 (1.033 to 1.166)	0.003	1.077 (0.980 to 1.613)	0.21
AH	1.520 (1.111 to 2.509)	0.04	1.542 (1.042 to 2.931)	0.03
CHD	1.429 (1.271 to 3.144)	0.002	3.001 (0.200 to 6.012)	0.24
**Southern Egypt**				
Age	1.011 (0.909 to 1.380)	0.04	2.110 (0.991 to 3.101)	0.31
AH	0.902 (0.400 to 1.970)	0.03	0.809 (0.106 to 2.121)	0.08
Obesity	1.901 (1.001 to 3.122)	0.01	2.990 (1.202 to 3.015)	0.02

MAE: major adverse events (death, re-infection, mechanical ventilation). Not enough data concerning clinical outcome in Europe and USA.

## Data Availability

Not applicable.

## Supplementary Materials

The following are available online at https://www.mdpi.com/article/10.3390/jcm10132752/s1, Figure S1: Influence analysis (without dioceses with high prevalence disease) of relationship between risk factors and prevalence of COVID-19 among clergy, Figure S2. Prevalence of SARS-CoV2 among priests in different diocese.
