# Supplementary material for: Obesity Strongly Predicts COVID-19-Related Major Clinical Adverse Events in Coptic Clergy
Source: J Clin Med. 2021 Jun 22;10(13):2752. doi: 10.3390/jcm10132752 (PMC8269321; doi:10.3390/jcm10132752)
Supplement: Supplementary file 1 [file jcm-10-02752-s001.zip › jcm-1222681-supplementary.pdf]

Supllementary Materials

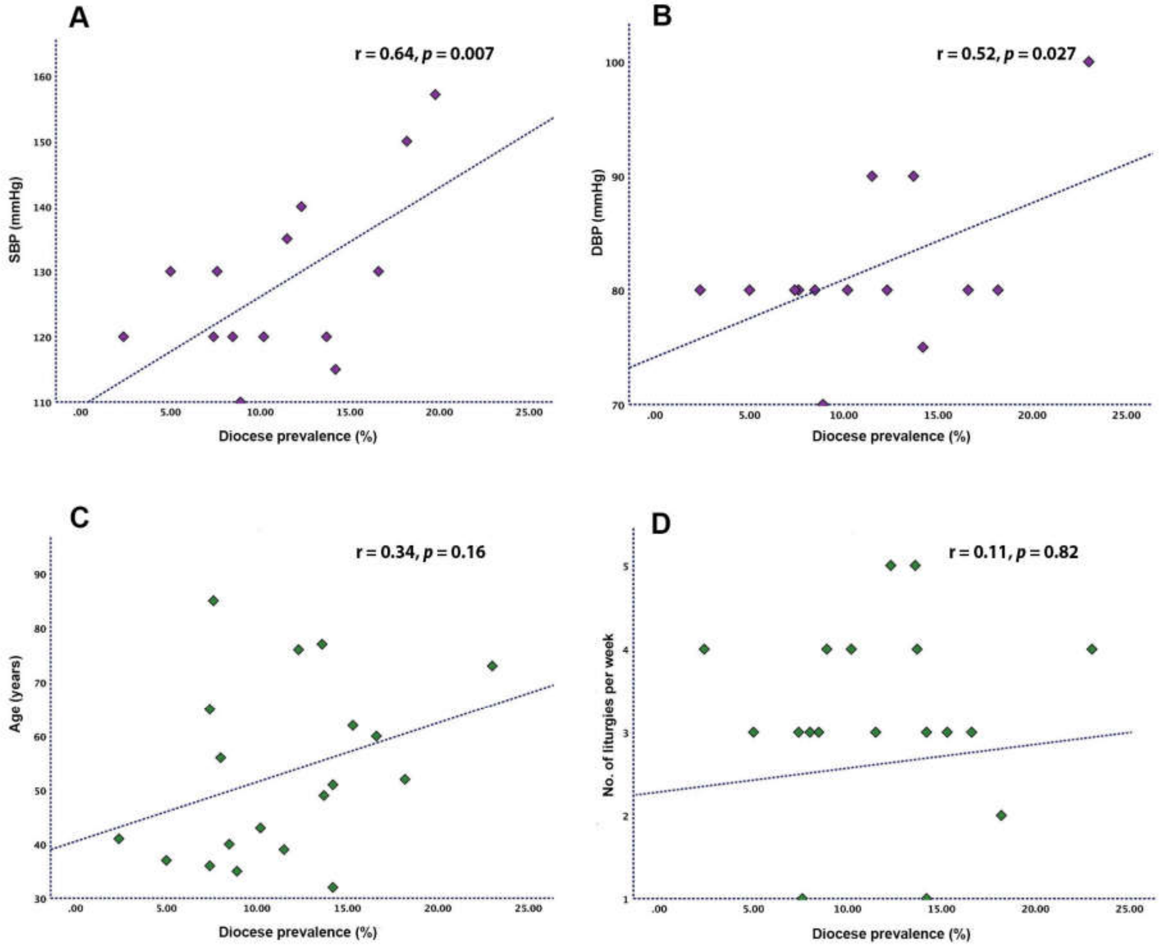

Figure S1. Influence analysis (without dioceses with high prevalence disease) of relationship between risk factors and prevalence of COVID-19 among clergy. (**A**) Prevalence disease with SBP; (**B**) prevalence disease with DBP; (**C**) prevalence disease with age; (**D**) prevalence disease with number of liturgies per week. SBP: systolic blood pressure; DBP: diastolic blood pressure.

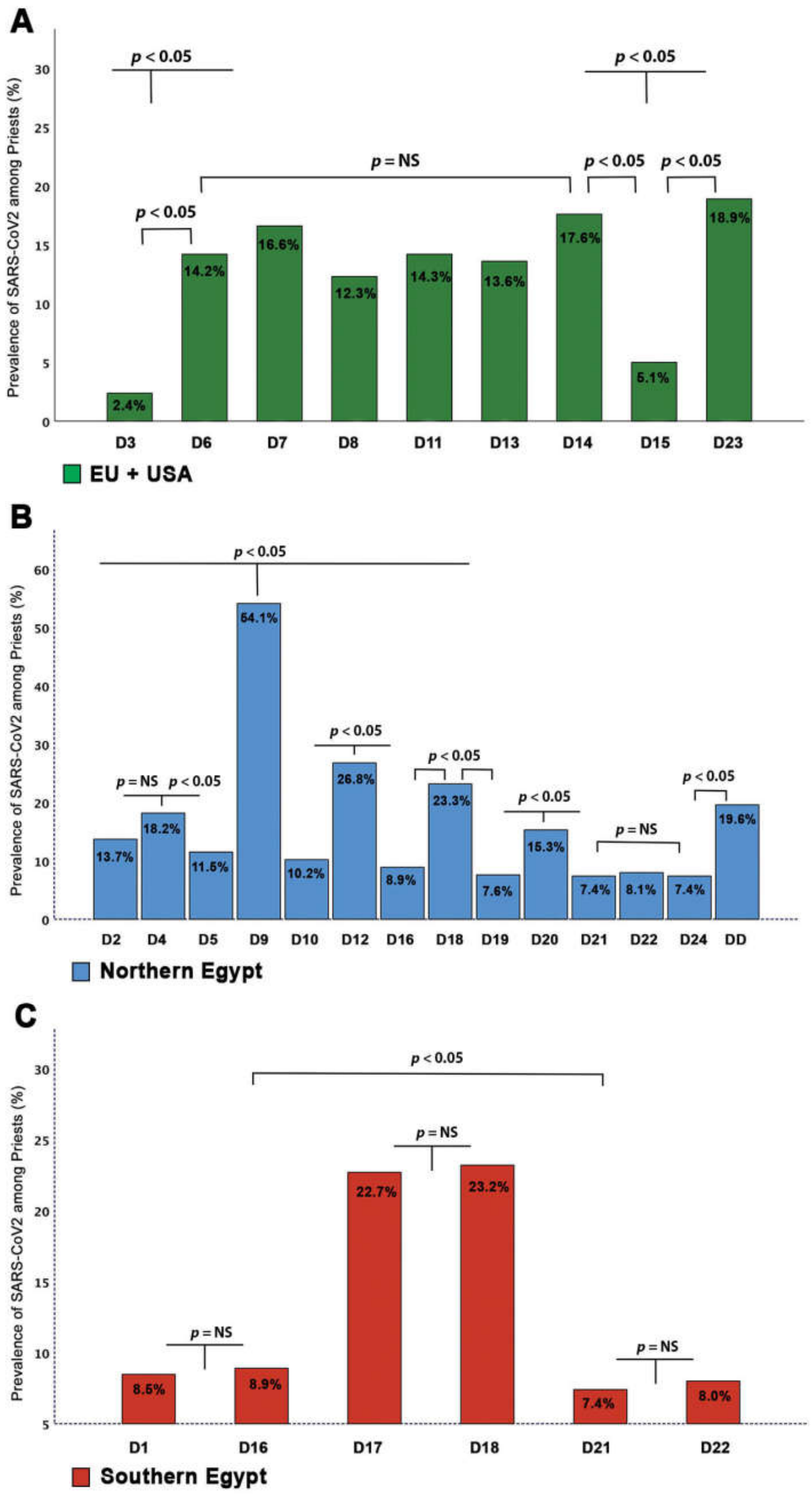

Figure S2. Prevalence of SARS-CoV2 among priests in different diocese; (**A**) EU + USA; (**B**) Northern Egypt; (**C**) Southern Egypt.
